# Asthma and Seroconversion from* Toxocara* spp. Infection: Which Comes First?

**DOI:** 10.1155/2018/4280792

**Published:** 2018-05-14

**Authors:** Paula Mayara Matos Fialho, Carlos Roberto Silveira Correa, Susana Zevallos Lescano

**Affiliations:** ^1^Departamento de Saúde Coletiva, Faculdade de Ciências Médicas, Campinas, SP, Brazil; ^2^Universidade de São Paulo, Instituto de Medicina Tropical de São Paulo, Laboratório de Imunopatologia da Esquistossomose (LIM 06), São Paulo, SP, Brazil

## Abstract

The aim of this study was to estimate the incidence of seroconversion of* Toxocara *spp. infection and related variables. We conducted a cohort study of 77 children aged 2–12 years who had negative serology in a previous cross-sectional study. Univariate and bivariate analyses were performed to describe the cohort, using socioeconomic, behavioral, and health conditions as variables. Logistic regression analysis was performed using seroconversion as the dependent variable, and the remaining variables are treated as independent variables. Asthma was the only independent variable that showed an association with seroconversion, with an odds ratio = 3.57 (1.01–12.6). The incidence of seroconversion from* Toxocara *spp. infection in the children followed was 10.4 per 100 per year. Previous studies reporting an association of asthma with toxocariasis have only been carried out using cross-sectional studies. Therefore, this study is one of only a few describing the incidence of seroconversion from* Toxocara *spp. infection, which is relevant for understanding the burden of this parasite.

## 1. Introduction

Toxocariasis is a cosmopolitan disease that occurs in various regions worldwide, more frequently in developing countries [1–4]. Owing to an increasing human population in large cities, with consequential increases in canine and feline populations, the feces of these animals are increasingly contaminating the urban environment. Therefore, toxocariasis is currently a common anthropozoonosis in developed countries, with a reported prevalence of 13.9% in the United States [5] and a range from 4.2% to 65.4% in Brazil [6].

Among the most commonly reported factors associated with toxocariasis is contact between humans and dogs [7, 8]. Asthma is described in various studies as a toxocariasis-associated factor [5, 8, 9]; however, the direction of this association is unclear. The definitive factors in this association must be clarified, and appropriate methodology is needed in order to further elucidate the relationship.

Various studies describe the prevalence of toxocariasis in humans [10–13]. However, its incidence is rarely studied unless a population sample is being described. Therefore, the current study aimed to estimate the incidence of seroconversion from* Toxocara *spp. infection as it related to a cohort of randomly selected children living in the city neighborhood of Campinas, São Paulo State, Brazil.

This study determined the incidence of seroconversion resulting from* Toxocara* spp. infection and tested several variables for association with this seroconversion. Asthma was the only variable that showed an association.

## 2. Materials and Methods

A closed prospective cohort study was carried out using a sample of children who had negative serology for* Toxocara *spp., and whose place of residence was in a neighborhood of São Marcos, in the Northern District of the city of Campinas, São Paulo, Brazil. The study was approved by the Research Ethics Committee of the Faculty of Medical Sciences, State University of Campinas, and Tropical Medicine Institute of the University of São Paulo.

**Figure 1:**
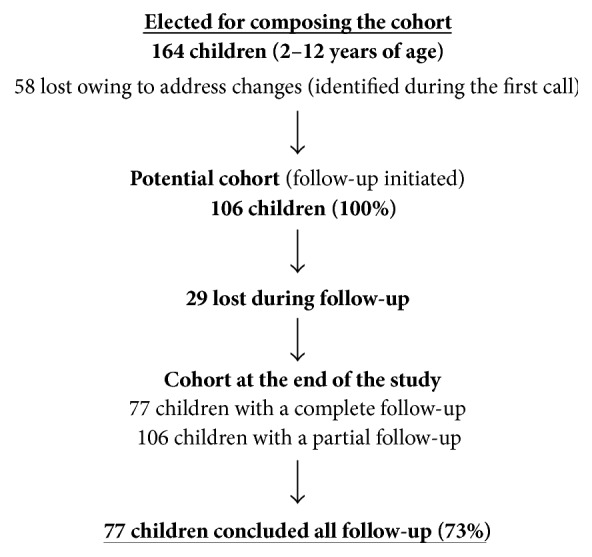
Cohort dynamics. Campinas, SP, Brazil, 2015.

The first stage was performed between 2012 and 2014 as part of a population-based study [6] comprising 200 children between the ages of 2 and 12 years. The study population was selected through a random sampling of houses registered in the Basic Attention Information and Management System (SIGAB, Sistema de Informação e Gerenciamento de Atenção Básica) of the Campinas Health Department. Initially, 192 children were evaluated for the presence of anti-*Toxocara* spp. antibodies, of which 28 presented with positive serology and were treated. The 164 who demonstrated negative serology against* Toxocara *spp. comprised the cohort of the current study. Because the cohort was open, 87 total losses were incurred (Figure 1). These losses were mainly caused by address changes (92%). The results of the current study are with reference to the 77 children that had completed all follow-up.

The cohort was followed over a 12-month period. Each child was visited four times, and all were asked about the presence of a rash and whether they had any respiratory complaints. Questions were formulated following a pretest performed with a limited number of individuals. The questions were designed to correspond with the International Study of Asthma and Allergies in Childhood (ISAAC) program. The questions asked during the evaluations are listed in Table 1.

### 2.1. Blood Samples and ELISA

One year after their last serological test, the children of the cohort underwent a new serological test for the diagnosis of IgG anti-*Toxocara *spp. antibodies. Blood collection from each child was performed using digital puncture and filter paper (Whatman™ Grade number 3) [14]. The collected samples were stored in notebooks, previously prepared using 1 cm wide filter paper strips separated by cellophane strips and filed according to the sequence of the sample collection. The child's ring finger was disinfected with 70% alcohol and the fingertip pierced using a disposable lancet. Blood was collected by sliding the ribbon filter paper on the finger until it was completely absorbed. The strips were dried at room temperature and stored at −20°C [6]. For processing, the blood samples were sent to the laboratory, eluted, and subjected to analysis using enzyme-linked immunosorbent assays (ELISAs) to detect IgG class antibodies using* T. canis* excretory-secretory (TES) antigen as a capture antigen. This technique has been previously described by de Savigny [15] and subsequently modified by Bach-Rizzatti [16].

#### 2.1.1. Preparation of the Eluate

Pieces of Whatman #3 filter paper were cut to size (1.0 cm^2^) from strips containing the blood collected from the fingertips of the study participants. The filter paper and 330 *μ*L of PBS-0.01, pH 7.2, were added to Eppendorf microtubes and incubated at 4°C for 16 hours.

#### 2.1.2. ELISA Plate Preparation

The reaction was performed in flat bottom 96-well polystyrene microplates (Costar 3590, high binding). The wells were coated with 100 *μ*L TES antigen solution diluted in PBS (10 *μ*g/mL). The plates were covered and incubated at 37°C for 2 hours, followed by 16 hours at 4°C. Prior to use, the nonspecific sites were blocked with 200 *μ*L of PBS-Tween-Gelatin (0.05%) for one hour at 37°C.

#### 2.1.3. Adsorption of Eluates

The eluates and control sera were adsorbed with total* Ascaris suum* antigenic extract to avoid cross-reactions with common* Ascaris* antigens.* Ascaris suum* antigen was diluted 1 : 200 in PBS-Tween to prepare an adsorption solution. The positive and negative control sera were diluted 1 : 320 in the adsorption solution (PBS-Tween-*Ascaris*) and incubated as with the eluates.

After blocking, the wells of the ELISA plates were washed three times with PBS-Tween (5 min each). Subsequently, 100 *μ*L of the preadsorbed eluates were added to the wells, in duplicate, and 200 *μ*L of known positive-patient sera were preabsorbed. In the subsequent rows, 100 *μ*L of PBS-T was put into each well, and 100 *μ*L from the first set of wells was passed using a multichannel pipette to the following rows, resulting in twofold serial dilutions. The last 100 *μ*L was discarded. Subsequently, 100 *μ*L of standard negative sera, absorbed with PBS-T-*Ascaris*, as well as two blanks with PBS-T, were placed into the first six wells. The plate was incubated for 40 minutes at 37°C. Further, the plate was washed three times with PBS-T. The enzyme conjugate anti-IgG labeled with peroxidase was diluted 1 : 5000 in PBS-TG and applied to all the wells and incubated at 37°C for 40 minutes. Another wash cycle was performed as previously described. Visualization was carried out by adding 100 *μ*L of an orthophenylenediamine solution in citrate phosphate buffer, with 5 *μ*L of 30% hydrogen peroxide (H_2_O_2_) and incubated in the dark at room temperature for 20 minutes. The reaction was stopped by adding 50 *μ*L of 4 N H_2_SO_4_ solution. The plates were read using a Multiskan™ ELISA reader with 492 nm filter. The cut-off density-point in the serological assays varied from 0.330 to 0.390 and was determined for each test using the mean optical density of 30 sera from the negative control group plus two standard deviations [6].

### 2.2. Statistical Analyses

Data analysis initially included the cohort description according to each variable studied, calculating absolute and relative frequencies, and their respective 95% confidence intervals (CI). A bivariate analysis was then performed for socioeconomic, behavioral, and health conditions as variables as a function of seroconversion from* Toxocara* spp. infection. Fisher's exact test was performed as indicated. When the association had *p* > 0.20, it was included in the regression model.

Logistic regression was performed, considering the presence of* Toxocara *spp. infection as a dependent variable, and the remaining variables as independent. The Forward Euler method was used, where one variable at a time was added in sequence, starting with the variables presenting a higher correlation value with the response variable. The logistic regression coefficient was raised to the power of “e” (Euler's number) in order to obtain the odds ratio (OR) of the association. The critical limit adopted for the test was 10% [17]. All statistical analyses were performed using SAS 9.4v and Epi Info 7 software.

## 3. Results

The results of the present study are based on 77 children who had negative serology in a previous cross-sectional study. The incidence of seroconversion from* Toxocara* spp. infection in this cohort was 10.4% (8/77; 10.4 cases per 100 children) per year.  Table 2 summarizes the descriptors for the children in the cohort who had completed the entire follow-up (*n* = 77). The mean age of the children was 7.7 years (range 2–12) with 63.6% of the children being male.

Results of the bivariate study of the cohort according to whether toxocariasis serology was positive are presented in Table 3. In the current study, we were unable to identify any association between the seroconversion from* Toxocara* spp. infection and socioeconomic, behavioral, or health-condition variables. Logistic regression analysis showed that asthma was a risk factor for seroconversion from* Toxocara* spp. infection with an OR of 3.57 (CI: 1.01–12.6). The remaining variables such as the type of property border, the number of residents in the home, and contact with dogs or cats did not present as statistical significance risk factors associated for seroconversion (Table 4).

## 4. Discussion

The data from our study indicate that the incidence of seroconversion from* Toxocara *spp. infection was 8/77 per year for the children that were followed-up or 10.4 per 100 children per year. This incidence was not significantly different from the one found by Correa and Bismarck [18] in the same territory. Subsequent to the study by Correa and Bismark, there has been an increase in the number of houses with sanitation, and also additional asphalt paving. This reinforces the cosmopolitan character of this parasitosis. Although this parasitic infection depends on the presence of geohelminth eggs in the environment, canine or feline contact was not an independent variable associated with this infection, possible owing to the small sample size. Therefore, other factors must be considered as they relate to the way in which a child may come into contact with these geohelminth eggs.

Among the evaluated independent variables, only the presence of asthma was found to be a risk factor for a positive serologic result for infection by* Toxocara*. The association between asthma and toxocariasis has been previously reported in various cross-sectional studies [8, 9, 19], which present an association between these two variables. However, in these types of studies it is not possible to conclude which condition is chronologically preceded by the other. In the current cohort study, we found that the presence of asthma preceded seroconversion from* Toxocara* spp. infection; thus, asthma preceded the serologic shift. One explanation is that there is a period of time between infection and the serologic shift, with the respiratory symptoms initiating prior to the serologic shift. Thus, asthma would be identified, not only as a disease, but also as an “unfavorable conditions* proxy*” that facilitates infection by the parasite, which is related to the seroconversion.

Several articles have previously identified the association of asthma with the presence of antibodies against* Toxocara* spp. The current study, based on the adopted model, allowed us to identify asthmatic children who presented with anti-*Toxocara* antibodies. This result suggests a factor, probably related to inflammation, that would be present in asthmatic children and that could facilitate them to seroconvert. Several published reports suggest that allergic manifestations, such as asthma, may be a consequence of parasitic infections [5, 8, 20, 21]. For more than two decades the hygienist theory has suggested that the increase in allergic diseases is a consequence of the decrease in parasitic infections [22, 23].

In the current work, using a cohort study, the results were contradictory. We observed that asthmatic children developed anti-*Toxocara* antibodies. These data, however, may be explained by an observation made by Maizels [23] in which the inflammatory response triggered by a parasite varies depending whether the host is the definitive or paratenic host of the parasite. In the case of* Toxocara*, the human is a paratenic host, and thus the inflammatory response is expected to be different from that triggered by another parasite of which the human being is the definitive host, which is the case for* Schistosoma mansoni* [24].

However, it is not possible to avoid the association of the data from this study with those obtained by Poorisrisak et al. [25]. While studying the association of asthma with infection by respiratory syncytial virus (RSV) in monozygotic twin infants, they found that asthma susceptibility influences infection by RSV. Data found in this study suggest that the presence of asthma may be a marker for whom would be infected by* Toxocara canis* or, at least, present antibodies for this parasite. Other children may be infected without producing antibodies. However, these conclusions should be considered with caution, because at this point, the data only suggest hypotheses to be tested in subsequent studies using a larger number of children involved.

The average age of our cohort was 7.7 ± 3.9 years, which is consistent with the age of participants in other studies in which higher toxocariasis prevalence was found [26]. Anaruma Filho et al. [13] found for the same region that the type of property border was a factor that contributed to the prevention of infection by* Toxocara*. This protection may be associated with the fact that obstacles such as walls or fences may limit access of dogs to the peridomicile. However, this association was not found in our study, possibly because of the fact that almost every house of both infected and noninfected children presented property borders such as gates, walls, and fences. Therefore, we could not discriminate between the hosts infected and not infected by the parasite. There are no epidemiological differences that reinforce the claim that the parasite is cosmopolitan and adapts to the social conditions of the territory.

 Fan et al. [27] observed that a high prevalence of toxocariasis occurred similarly in individuals who had contact with dogs and those who had not and thus suggested that both groups present the same risk for infection by* Toxocara canis*. It is important to highlight that various parents/guardians of the children reported that they did not have dogs or cats at the home, but that the child did have daily contact with these animals at the homes of their relatives.

A mother's level of education was another variable that was not statistically significant, which contrasts the findings of Ferreira et al. [28] who attributed a significant decrease in the prevalence of enteroparasitosis in children living in the city of São Paulo, Brazil, to the mother's level of education and to the income improvement observed over the last decades. However, it is worth noting that this was a study conducted in a neighborhood of Campinas, Brazil, that has a low human development index (HDI), and where 25% of pregnant women are younger than 20 years of age and on average have a lower level of education [29].

An important factor in the current study is the significant number of children who abandoned the cohort. This loss occurred exclusively because of families moving from the study region, probably because of social factors that forced their relocation. Such a significant loss was unexpected; however, these losses did not compromise the findings or the impact of the work.

It is also important to note that access to families and the territory where they lived was assessed with the knowledge and consent of those responsible for the region's Primary Care Unit and was facilitated by the community health agent responsible for that location. The cohort was assessed during the same time period as the largest dengue outbreak in Campinas' history, which brought an important outcome to this study.

The diagnosis of toxocariasis is performed through antibody detection. Various techniques have been developed, with the most commonly used being ELISA in which larval excretory-secretory products are used [30]. The current ELISA being used has a 78% sensitivity and a 92% specificity [31]. This makes it possible to identify truly positive or truly negative individuals using these data, since a sensitive test rarely fails to detect individuals inflicted with the disease, while a highly specific test will rarely incorrectly categorize individuals who do not have the disease.

It is important to emphasize in this discussion that the presence of detectable anti-*Toxocara* antibodies does not necessarily indicate an active infection. False-positive reactions may occur in individuals suffering from ascariasis, schistosomiasis, or filariasis. It was not feasible to perform a geohelminth survey during our study because of the low adherence by the cohort in providing fecal samples for parasitology tests.

The majority of children infected with* Toxocara* are asymptomatic. However, as a result of the overall number of infected children, the development of more severe clinical manifestations is possible, which may compromise their quality of life. In general, the awareness by health professionals, government workers, and educators with respect to toxocariasis as a public health problem is both necessary and urgently needed. It is also important that preventive and educational measures are employed in order to control this parasitosis.

## Figures and Tables

**Table 1:** Questions asked during each follow-up visit of the cohort regarding the presence of rashes and any respiratory complaints. Campinas, SP, Brazil, 2015.

(1) “Has your son or daughter experienced a cough, shortness of breath, or wheezing during the last month?”
(2) “Has your son or daughter visited any health-service provider owing to respiratory problems during the last month?”
(3) “Has any doctor told you that your son or daughter has or had asthma?”

**Table 2:** Description at the start of the study (*T*0^a^) of the children (*n* = 77^b^) in the cohort that completed follow-up, relative to the characteristics considered in the study. Campinas, SP, Brazil, 2015.

Variable	*n*	%	95% CI^c^
*Toxocariasis (seropositive)*			
Yes	0	0	
No	77	100	95.32–100.00
*Property borders*			
Walled	67	92	82.96–96.92
Fenced	2	3	0.33–9.55
None	4	5	1.51–13.44
*Number of residents in the home*			
<6	66	10	81.24–96.06
>6	7	90	3.94–18.76
*Contact with dog(s)*			
Yes	59	81	69.92–89.10
No	14	19	10.90–30.08
*Contact with cat(s)*			
Yes	31	42	30.97–54.59
No	42	58	45.41–69.03
*Asthma*			
Yes	18	24	14.89–32.25
No	57	76	64.75–85.11

^a^ *T*0 = time 0; ^b^*n* = number of participants. ^c^Confidence interval.

**Table 3:** Bivariate analysis of the 77 children in the cohort regarding seroconversion from *Toxocara *spp. infection and independent variables. Campinas, SP, Brazil, 2015.

Variable	Seroconversion of *Toxocara *spp.					*p* ^*∗*^
	Yes			No		
	*n*	%		*n*	%	
*Property borders*						
Walled	8	11.0		59	80.8	0.48
Fenced	0	0.0		2	2.7	
None	0	0.0		4	5.5	
*Number of residents in the home*						
<6	6	8.2		38	52.1	0.21
>6	2	2.7		27	37.0	
*Contact with dog(s)*						
Yes	7	9.6		1	1.4	0.35
No	52	71.2		13	17.8	
*Contact with cat(s)*						
Yes	5	6.8		3	4.1	0.14
No	26	35.6		39	53.4	
*Asthma*						
Yes	4	5.6		4	5.6	0.08
No	14	19.4		50	69.4	

^*∗*^Fisher's exact test.

**Table 4:** Logistic regression model^a^ for the 77 children of the cohort with seroconversion from *Toxocara* spp. infection. Campinas, SP, Brazil, 2015.

Variable	Odds ratio	90% CI^b^
Asthma	3.57	1.01–12.6

^a^Forward Euler method of selection. ^b^Confidence level.
